# Genomic identification of expressed globulin storage proteins in oat

**DOI:** 10.3389/fpls.2024.1418658

**Published:** 2024-07-23

**Authors:** Aina Belén Gil-González, Lars L. E. Sjögren, Katja Bernfur, Olof Olsson, Jose Alfredo Zambrano

**Affiliations:** ^1^ Division of Pure and Applied Biochemistry, Department of Chemistry, Lund University, Lund, Sweden; ^2^ Plant Breeding, Lantmännen, Svalöv, Sweden; ^3^ Division of Biochemistry and Structural Biology, Department of Chemistry, Lund University, Lund, Sweden

**Keywords:** Avena sativa, cereal proteins, globulins, mass spectrometry, Osborne fractionation, protein isolation

## Abstract

**Introduction:**

Oats, a highly nutritious cereal known for their health benefits, contain various macromolecules of significant biological value, including abundant and highly digestible proteins. Despite their importance, oat proteins have not been extensively studied. Here, we present a complete set of the expressed globulins genes, which code for the main storage protein in oats as well as their chromosomal positions.

**Methods:**

Published expressed sequence tags for globulins were used as queries in the Sang oat genome. In addition, globulin proteins were fractionated from oat flour by solvent extraction based on differential solubility with other classes of cereal proteins. The protein fractions were separated by gel electrophoresis and analyzed by tandem mass spectrometry to confirm their identity and expression in seed.

**Results and discussion:**

In total 32 globulin gene sequences were identified on the oat genome. Out of these, the expression on RNA level could be confirmed and 27 were also detected as expressed proteins by MS. Our results provide the most extensive set of salt-soluble oat globulin sequences to date, paving the way for further understanding their implications for human nutrition. In addition, a simple methodology to fractionate oat proteins is presented.

## 1 Introduction

Oats (*Avena sativa*) have been cultivated by humans since 1000 BC and have traditionally been used for both food and feed (Menon et al., 2016). During the last 15 years, oats have become an important part of contemporary health-conscious lifestyles, especially in Western countries. Presently, over 25 million tons of oats are harvested every year, which corresponds to 2% of the global grain harvest (FAO, 2023). Especially oat dietary fibers and proteins are of great interest, but this crop also contains unsaturated fatty acids, vitamins, avenanthramides, and other nutrients (Spaen and Silva, 2021). A regular intake of oat and derived products contributes to human health, as recognized in several health claims issued by regulatory authorities such as the Food and Drug Administration (FDA) in the USA or the European Food Safety Authority (EFSA) (Food and Drug Administration, 1998; EFSA Panel on Dietetic Products Nutrition and Allergies, 2010, 2011a, 2011b).

Oat grains contain about 60% of starch, which makes it the most abundant carbohydrate. However, they are also rich in dietary fibers, which are of particular interest since they contribute to numerous physiological benefits in both animals and humans (Antonini et al., 2016). Whole oat grains are reported to have between 20 and 37% dietary fiber, with β-glucan as the predominant one (Menon et al., 2016). When consumed, the β-glucan fibers create viscosity in the intestine, which lowers glucose absorption and the insulin response by modulating starch digestion, as well as preventing cholesterol reabsorption (Braaten et al., 1994a; Braaten et al., 1994b; Paudel et al., 2021). This is beneficial in general, but especially positive for type II diabetics and cardiovascular disease patients.

The protein content of oat kernels ranges from 12 to 20%, depending on the variety and cultivation conditions. Thus, oat is one of the food crops with the highest protein concentrations (Menon et al., 2016; Poutanen et al., 2022). Oat proteins are generally regarded as safe also in gluten-free diets because of the weak immunological activities of avenins (Ahola et al., 2020). The actual protein composition, based on the Osborne classification, differs from other cereals. The principal fraction in oats is globulins (70 – 80%), followed by prolamins (avenins; 4 – 14%), albumins (enzymes, 1 – 12%), and a minor fraction of glutelins (<10%) (Antonini et al., 2016; Spaen and Silva, 2021).

Globulins can be classified according to their different sedimentation coefficients, that is, 3S, 7S, and 12S (Mäkinen et al., 2017). The 12S proteins are composed of two disulfide bridge-linked polypeptides arising from a single post-translational cleaved protein (Peterson, 1978; Brinegar and Peterson, 1982). These are denoted α and β have molecular weights (MW) around 22 and 32 kDa, respectively. The linked subunits assemble and form a predominant hexamer quaternary structure (Zhao et al., 2004).

Oat globulins have a relatively higher lysine content than the other protein classes mentioned above, and this is the main limiting essential amino acid in most grains (Paudel et al., 2021). Since globulins are the major storage proteins in oats, this cereal has protein with a higher biological value compared to, for example, wheat and maize where prolamins are dominant (Mel and Malalgoda, 2022). In addition, oat proteins are considered of better quality compared to other crops due to their higher digestibility and abundance (National Research Council, 1989). The digestible indispensable amino acid score (DIAAS) is used as a measure to define good protein sources for human consumption, and based on the cut-off value from FAO, oats are considered among the best grain protein sources (Leser, 2013; Cervantes-Pahm et al., 2014). The protein efficiency ratio (PER) is also higher in oats than in other cereals, and therefore, oat proteins can be efficiently utilized by the human body (Tang et al., 2023). Moreover, bioactive oat peptides with several positive regulatory activities can be produced through enzymatic hydrolysis (Rafique et al., 2022). Overall, oat proteins are an excellent choice to enhance human well-being due to their nutritional quality and beneficial properties.

Research on oats has been predominantly focused on dietary fibers, due to the well-documented health benefits associated with them. However, it is essential to extend the knowledge to oat proteins, since they make a positive contribution to human nutrition and could be an asset of plant-based high-protein foods. These plant-based products have been proven to be the way to go to reach United Nations’ sustainable development goals (SDG) with an emphasis on SDG #2 (ending global hunger) and SDG #12 (achieving sustainable consumption and production) (Saari et al., 2021).

A better understanding of oat proteins abundance, expression and potential properties will pave the way for future advancements in human nutrition. This is especially important for globulins, as the major protein component.

The most extensive available description of the oat globulins identified 24 unique RNA sequences from expressed sequence tags (EST) for the oat cultivar CDC Dancer (Anderson, 2014). These EST represented 16 full and 8 partial sequences. However, when the cited study was performed, the oat full genome had not yet been published, making it impossible to obtain a complete set of globulin genes. The large hexaploid genome of oat (ca 11 Gb) has significantly slowed down complete sequencing. In 2022 the annotated genome for the cultivar Sang was published, revealing high complexity that is underscored by its extensive repeats and major genomic rearrangements, including 7 large-scale translocations and transposable elements accounting for 64% of the genome (Kamal et al., 2022). The access to the genome sequence enables researchers to acquire a deeper and more precise knowledge of the fundamental genetics of oats, laying the foundation for basic research and accelerating the emergence of novel applications (e.g., developing improved oat varieties with enhanced nutritional profiles or resilience to environmental stresses).

In the present study, we retrieved and mapped the expressed globulins in oats to gain a comprehensive understanding of the most abundant protein family in this cereal. Building on previously documented gene sequences, we searched for homology by employing our recently developed Sang genome browser (Kamal et al., 2022). These sequences were compared with the re-sequenced and reference-assembled Belinda oat genome. A set of 32 globulin genes in both genomes was retrieved, out of which 27 were confirmed to be expressed on protein level. To do so, oat globulin proteins were fractionated according to solubility (Osborne), separated by gel electrophoresis, and identified using tandem mass spectrometry. This paves the way for a better characterization of oat proteins with future perspectives on improving breeding programs to achieve more nutritious varieties.

## 2 Materials and methods

### 2.1 Oat samples and sample preparation

Oat groats from two independently grown field trials of the cultivar Belinda were used in the experiments. The oats were grown in Lönnstorp, Sweden, year 2020 and 2021 and were denoted as LT20-Belinda and LT21-Belinda.

Approximately 5 g of the harvested seeds were manually dehulled and milled to flour using the Fritsch™ Pulverisette 23 Mini Mill (Fisher Scientific, Leicestershire, UK) instrument with the following settings: 50 oscillations (50 Hz) for 2 min, 2 cycles, 4 min of total milling time. The flour was stored at -20°C in Falcon tubes to conserve the proteins.

### 2.2 Gene annotation and phylogenetic analysis

A total of 24 globulin EST originating from CDC Dancer oat cultivar (Anderson, 2014) were used as a query in Sang Genome Browser to identify similar genes (Kamal et al., 2022). Annotation data and associated analyses for *A. sativa* cv. Sang are from GrainGenes (Blake et al., 2019): Sang genome browser, https://wheat.pw.usda.gov/jb/?data=/ggds/oat-sang; Sang data download, https://wheat.pw.usda.gov/GG3/content/avena-sang-download. The globulin protein sequences were translated and the MWs were obtained using the Protein Molecular Weight program in The Sequence Manipulation Suite (Stothard, 2000).

Multiple sequence alignment (MSA) of the 32 obtained sequences was performed with Clustal Omega, default settings (Sievers et al., 2011). Visualization of the alignment was run in ESPripr 3.0 with a score of 0.7 out of 1 to show equivalence based on amino acid physicochemical properties (Robert and Gouet, 2014).

The evolutionary history was inferred using the Neighbor-Joining method (Saitou and Nei, 1987). The bootstrap consensus tree was inferred from 1000 replicates (Felsenstein, 1985). Branches corresponding to partitions reproduced in less than 50% bootstrap replicates were collapsed. The percentage of replicate trees in which the associated taxa clustered together in the bootstrap test (1000 replicates) are shown next to the branches. The evolutionary distances were computed using the Poisson correction method and are in the units of the number of amino acid substitutions per site (Zuckerkandl and Pauling, 1965). This analysis involved 32 amino acid sequences. All ambiguous positions were removed for each sequence pair (pairwise deletion option). The final dataset consisted of 531 positions. Evolutionary analyses were conducted in MEGA11 (Tamura et al., 2021).

### 2.3 Protein extraction from flour

#### 2.3.1 Total protein extraction

250 µL of ultra-pure deionized water were added to 40 mg of oat flour from LT20-Belinda in a 2 mL tube containing a 6 mm glass bead, followed by thorough mixing using a Precellys^®^ machine (Bertin Technologies SAS, Montigny-le-Bretonneux, France) at 4500 rpm for 4 times x 60 s. Protein was extracted from the resulting slurry by adding 250 µL of 2X Laemmli sample buffer containing ß-mercaptoethanol. The samples were heat treated at 70 °C for 10 min in a heat shaker at 1200 rpm to prevent the flour from sinking during the extraction. Samples were then spun at 10000 g for 10 min at +4°C to pellet debris after which 200 µL from each of the different supernatants were carefully collected and moved to new Eppendorf tubes stored on ice. Finally, the samples were further diluted 4 times with 1X Laemmli sample buffer containing bromophenol (Bio-Rad Laboratories, Inc., Hercules, USA).

#### 2.3.2 Sequential protein extraction (Osborne fractionation)

Flour samples were defatted with hexane (1:3 w/v; Sigma-Aldrich, Saint Louis, USA) for 30 min in an orbital shaker. The procedure was repeated 3 times. After this, proteins were sequentially extracted by transferring 100 mg of the defatted flour to 2 mL tubes containing a 6 mm glass bead. 1 mL of extraction solution (detailed below) was added, and the contents were mixed using the Precellys^®^ machine (4500 rpm, 2 times x 15 s). Next, the extract was incubated in an orbital shaker with the following extraction solutions, time and temperature settings according to each fraction. Finally, the samples were centrifuged at 20000 g for 15 min at 4°C and the supernatants were collected. After each extraction, the pellet was washed 3 times x 15 min with the same extraction solution.

The extraction solutions were the following. For water-soluble proteins, ultra-pure deionized water was used for 1 h at 4°C. For salt-soluble proteins, a salt buffer (1 M NaCl, 50 mM Tris-HCl pH 8.5) was used for 3 h at 4°C. For alcohol-soluble proteins, 70% ethanol was used for 2 h at room temperature. For glutelin-like proteins, a 2% SDS, 10 mM NaOH buffer was used for 1 h at room temperature.

This procedure was followed most of the time as indicated and was referred to as the “standard protocol”. However, in some cases, based on differences in the extraction efficiency as visualized during gel electrophoresis, additional extractions and other minor modifications were made.

For albumin extraction, a 50 mM Tris-HCl pH 8.5 solution was employed for comparison (reported as “Tris protocol” later in this report and figures). Additionally, to determine if the extraction solution was saturated with globulins, preventing their complete extraction in the first step, an extra step consisting of a repetition of the second step was carried out.

#### 2.3.3 Protein visualization

Sodium dodecyl sulfate–polyacrylamide gel electrophoresis (SDS-PAGE) was performed by loading 20 μL (ca 5-50 μg protein depending on fraction) of each sample into 8–16% Criterion™ TGX gels (Bio-Rad Laboratories, Inc., Hercules, USA) with 1X tris/glycine/SDS running buffer. It was run at 80 mV for 2 hours and it was then stained with Bio-Safe™ Coomassie Stain (Bio-Rad Laboratories, Inc., Hercules, USA) for 1 hour, and distained with ultra-pure deionized water overnight. Precision Plus Protein Dual Color Standards (Bio-Rad Laboratories, Inc., Hercules, USA) was used as a molecular marker.

### 2.4 Mass spectrometry

#### 2.4.1 Sample preparation

Protein samples were obtained from the polyacrylamide gels. Four gel bands were cut. Each gel band was cut into 1 × 1 mm gel pieces, destained and further digested as follows. The gel pieces were incubated for 3 times x 30 min in 50% acetonitrile (ACN, Sigma-Aldrich, Saint Louis, USA)/50 mM ammonium bicarbonate (ABC). After this, the gel pieces were dehydrated using 100% ACN and then, the proteins were reduced with 25 µL 10 mM dithiothreitol (DTT) in 50 mM ABC buffer for 30 min at 37°C. The DTT was removed, and the gel pieces were again dehydrated in 100% ACN. The proteins were then alkylated with 25 µL 55 mM iodoacetamide in 50 mM ABC for 30 min in the dark at room temperature. The gel pieces were dehydrated a final time in 100% ACN before the proteins were digested by adding 25 µL of 12 ng/µL trypsin (sequence grade modified trypsin porcine, Promega, Fitchburg, USA) in 50 mM ABC buffer and incubated on ice for 4 h. Thereafter, 20 µL 50 mM ABC were added and the proteins were incubated overnight at 37°C to digest the proteins. The following day, the reaction was stopped by the addition of 10% formic acid (FA) to a final concentration of 0.5% at a pH of 2-3. Finally, the generated peptides were extracted and transferred into new tubes for analysis.

#### 2.4.2 Liquid chromatography-tandem mass spectrometry

Digested peptide samples were injected into an ultra-high pressure nanoflow chromatography system (nanoElute, Bruker Daltonics, Billerica, USA). The peptides were loaded onto an Acclaim PepMap C18 (5 mm, 300 μm id, 5 μm particle diameter, 100 Å pore size) trap column (Thermo Fisher Scientific, Waltham, USA) and separated on a Bruker Pepsep Ten C18 (75 µm × 10 cm, 1.9 µm particle size) analytical column (Bruker Daltonics, Billerica, USA). Mobile phase A (2% ACN, 0.1% FA) was used with the mobile phase B (0.1% FA in ACN) for 45 min to create a gradient (from 2 to 17% B in 20 min, from 17 to 34% B in 10 min, from 34 to 95% B in 3 min, at 95% B for 12 min) at a flow rate of 400 nL/min and a column oven temperature of 50°C. The peptides were analyzed on a quadrupole time-of-flight mass spectrometer (timsTOF Pro, Bruker Daltonics, Billerica, USA), via a nanoelectrospray ion source (Captive Spray Source, Bruker Daltonics, Billerica, USA) in positive mode, controlled by the OtofControl 5.1 software (Bruker Daltonics, Billerica, USA). The temperature of the ion transfer capillary was 180°C. A DDA method was used to select precursor ions for fragmentation with one TIMS-MS scan and 10 PASEF MS/MS scans. The TIMS-MS scan was acquired between 0.60–1.6 V s/cm^2^ and 100–1700 m/z with a ramp time of 100 ms. The 10 PASEF scans contained a maximum of 10 MS/MS scans per PASEF scan with a collision energy of 10 eV. Precursors with maximum 5 charges with intensity threshold to 5000 a. u. and a dynamic exclusion of 0.4 s were used.

#### 2.4.3 Data analysis

Raw data were processed using Mascot Distiller (version 2.8, Matrix Science, London, UK) and all data were searched using Mascot Daemon (version 2.5, Matrix Science, London, UK) against an in-house database containing the 32 amino acid sequences of the *in-silico* retrieved globulins. This targeted approach aligns with the objective of confirming the expression of the sequences, in contrast to using a large database with unrelated entries, which would be more suitable for broad proteomic discovery. Moreover, we seek to go beyond known entries already present in repositories to validate new globulins.

The following settings were applied: precursor ion tolerance 10 ppm; MS/MS fragment mass tolerance 0.015 Da; trypsin as protease; 1 missed cleavage site; and oxidation (M) as a variable modification and carbamidomethylation (C) as fixed modification. The significance threshold was set at p < 0.05 and the recommended ion score cut-off by the software was 25, which suppressed non-significant matches from the search.

## 3 Results

### 3.1 32 globulin genes are present in the oat genome

Globulin genes were first extracted by using the published globulin EST (Anderson, 2014) as queries to raw scaffold reads of the Sang oat genome. Gene annotation and expression were published in Kamal et al. (2022). A total of 29 high-confidence genes and 3 low-confidence genes were manually retrieved (**Supplementary File Data Sheet 1. FASTA**). For the current report, the obtained sequences were numbered for identification (e.g., “Globulin 1”, and the shortened version “Glo_1” for the phylogenetic tree, MSA and MSMS results).

High-confidence genes were defined as genes that met the following criteria: a) the presence of start and stop codons; b) well-defined splice sites; c) the presence of all exons expected from splice sites and their existence confirmed by RNA seq; d) the presence of a signal peptide; e) no unidentified nucleotides in the sequence (“N”). The three globulin genes classed as low confidence lacked the complete expression in RNA seq data (globulin 2), or the signal peptide (globulin 31) or the nucleotide sequence was not correctly deciphered (globulin 32).

**Figure 1:**
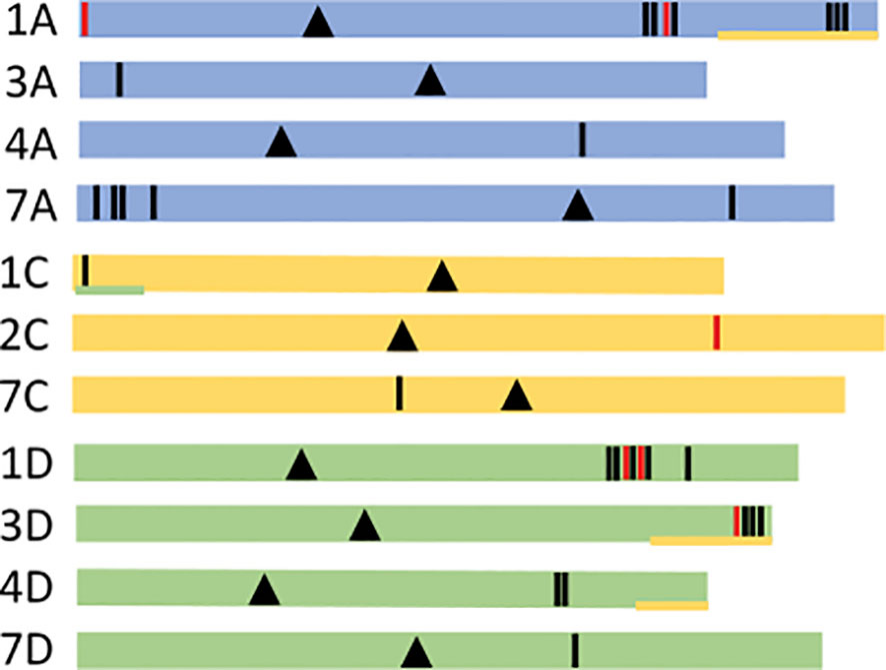
Chromosome localization of the globulin genes. Schematic figure of globulin chromosomal gene positions. Globulin genes are shown as vertical lines in the three different sub-genomes A, C and D, represented as colored lines (A blue, C yellow, D green). Black lines indicate forward (+) gene orientation and red reverse (-) orientation. Chromosomal centromeres are marked as black triangles. Ancestral sub-genome signatures are marked with an underlying line with corresponding sub-genomic color codes (Kamal et al., 2022). Exact chromosomal gene positions can be viewed in **Supplementary Table 1**.

Information on the genetic sequences is summarized in **Supplementary Table 1**, including the chromosome location, the gene and cDNA length. Physical chromosomal positions were determined by searching the Sang genome browser. Most of the globulin genes were correctly annotated in the gene browser but some were absent or incorrect. The identified sequences were found to be present in chromosomes 1A, 1D, 2C, 3A, 3D, 4A, 4D, 7A, 7C and 7D (**Figure 1**). Fifteen were located in the A-subgenome, 14 in the D-subgenome, and 3 in the C-subgenome. Only 6 are in the reverse orientation.

Additionally, the protein sequences were translated (**Supplementary File Data Sheet 2. FASTA**) and their amino acid length and total MW are reported considering the signal peptide and the α- and β-peptides. The smallest protein is 472 amino acids long and the longest one is 527. The average protein length is 508 amino acids, although only three of them are below 500. The calculated MW ranges from 53.35 to 59.36 kDa.

### 3.2 Protein phylogeny shows two major clusters

After obtaining the protein sequences, MSA was conducted to investigate similarities and differences among them. Moreover, a phylogenetic analysis was also performed.

The results displayed a high similarity and identity between all the sequences (**Supplementary Figure 1**). Only 109 of the residue positions analyzed were shown to maintain a similarity score lower than 70% for their physicochemical properties. Over 30% of the residues from the *α* subunit (88 out of 280 positions) have been found to be identical in all sequences. Identity for all proteins for the β subunit cannot be considered due to a premature stop codon in Globulin 2 in position 312, but the high similarity is maintained. The majority of the gaps and a higher proportion of less conserved residues were found between positions 260 and 300 of the MSA. This region was found to be a part of the β-subunit C-terminal. In addition, the last 15 residues of the α-subunit C-terminal were also found to have a higher variability. As previously mentioned, it can be observed that Globulin 31 lacked the signal peptide. Globulin 32, which was classified as low confidence, stands out as it shows a gap between the 60 and 110 positions, and a smaller one around 360.

**Figure 2:**
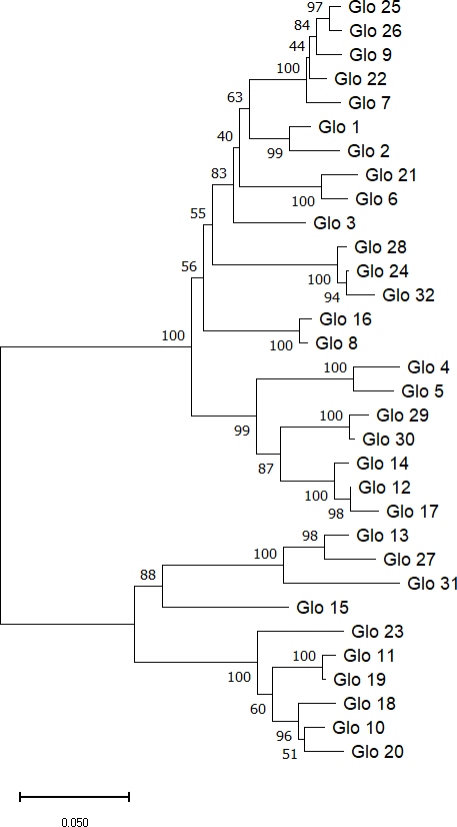
Phylogenetic tree of Sang globulin protein sequences. The tree was generated using the neighbor-joining method in MEGA11 software (Tamura et al., 2021). Next to the branches are the percentage of replicate trees showing each cluster in the bootstrap check (1000 replicates). Evolutionary distance of 0.05 was calculated with Poisson correction.

Taking advantage of the MSA data and employing the Neighbor-Joining method, the evolutionary history of the included sequences could be inferred. The resulting phylogenetic tree including bootstrap checks with 1000 replicates contains two major clusters (**Figure 2**) with 10 and 22 different sequences, respectively.

### 3.3 Globulin isolation by Osborne classification

The next step to continue the characterization of the oat globulins was to experimentally show that the protein sequences deduced from the genome are expressed. Expression can be tested at gene or protein level and in this study the latter was chosen. The major reason for this was that protein identification is more relevant since proteins represent the outcome of gene expression. Another reason was the difficulty in designing specific enough primers for quantitative polymerase chain reaction (qPCR) analysis due to the high similarity of the sequences. Thus, the protein extraction procedure was optimized for further peptide analysis with MS.

**Figure 3:**
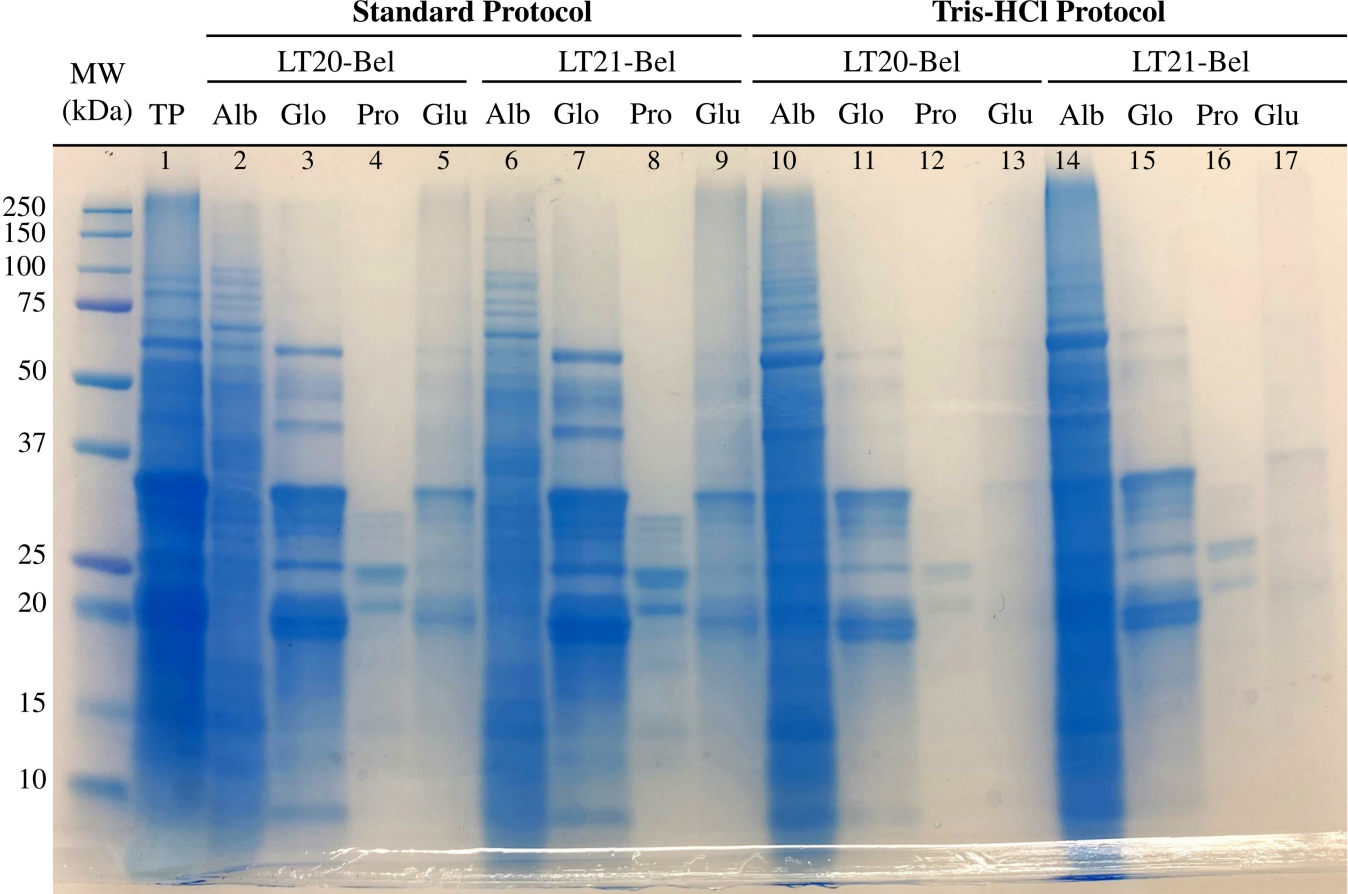
SDS-PAGE analysis of protein extracted from oat Belinda flour. Oat proteins (total protein – TP) and its fractions (albumin – Alb, globulin – Glo, prolamin – Pro, glutelin – Glu) were extracted by employing solvent solubilization. Samples from the Belinda cultivar and two different harvests were used (LT20-Bel and LT21-Bel). Two different solvents were used for albumin extraction: ultra-pure deionized water (Standard protocol) or 50 mM Tris-HCl pH 8.5 (Tris-HCl protocol). The gel used had a 8–16% polyacrylamide gradient and was stained with Coomassie. Molecular weight marker is shown as kDa on the left side.

Taking into consideration that cereal proteins can be separated based on their differential solubilities, the first step was to isolate each of the protein classes from the oat kernels by employing sequential solvent extractions. In our protocol, this resulted in four different fractions (i.e., water-soluble albumins, salt-soluble globulins, alcohol-soluble prolamins, and detergent-soluble glutelins with the remaining non-extracted proteins). The totality of proteins was also extracted. All samples were visualized by SDS-PAGE (**Figure 3**).

As can be observed from **Figure 3**, a wide range of proteins with different MWs ranging from 10 to 200 kDa are present in oat seeds (total protein – TP – lane 1). The two especially intense blue bands close to 20 and 35 kDa that are easily spotted are the 12S globulin subunits. The two first sets of lanes (lanes 2 to 9, under “standard protocol”) show the protein extracted by two Belinda samples using the standard protocol. Since the amount of protein loaded in the gel varies between samples, the intensity of the bands was not compared.

The first solvent extraction, representing the albumins (Alb, lanes 2 and 6) contained a large proportion of the total proteins. Some well-defined bands can be visualized but the general appearance is a smear. The globulin extraction (Glo, 3 and 7) resulted in more distinguishable bands at several MWs. The two main bands correspond to the 20 and 35 kDA subunits of the 12S globulin. The prolamin extraction resulted in two predominant bands between 20 and 25 kDa, although some less intense ones can be observed with similar MWs (Pro, lanes 4 and 8). The last lanes (Glu, 5 and 9) present a fainter smear with two bands, again in the same position as the 12S subunits.

To test differences with the solvent extraction for the water-soluble proteins, a low ionic tris-HCl buffer was employed instead of ultra-pure deionized water. The results are displayed in the right section of **Figure 3** (lanes 10 to 17, under “Tris-HCl protocol”). This led to higher protein extraction than in the first step, as the protein smear in the Alb lanes has a darker color than the equivalents from the standard protocol. Consequently, the following extractions exhibit fewer and fainter bands. Nevertheless, there is still a trace of the α- and β-globulin subunits in the Glu lanes, mainly seen in lane 17, which belongs to LT21 Belinda. This procedure was not found to be adequate for globulin isolation and was, thus, not employed for the following steps.

As observed following both protocols, bands for the 12S peptide subunits are present in the last step of the extraction, where a denaturing agent is used to extract all possible protein remnants. It was hypothesized that the high concentration of globulins saturated the solvent. To evaluate the theory, the standard protocol was repeated including an extra step, namely a second salt-soluble extraction that was performed consecutively after the first one.

The results showed empty lanes that correspond to the duplicated step, so no protein was extracted by following the procedure (**Supplementary Figure 2**). However, in the last lane, the corresponding 12S subunit bands were present. This result indicates the possibility that a fraction of the oat globulin proteins is non-salt soluble.

### 3.4 LC-MSMS identified 27 of the predicted proteins

The results obtained in the SDS-PAGE separations served as a sample source for peptide analyses with tandem MS. Four gel bands of MWs above 20 kDa and below 37 kDa were cut (**Supplementary Figure 3**), treated as described in Section 2, and digested with trypsin. Those bands were cut from both the globulin and the glutelin lanes. The reason for analyzing 4 samples was to compare the proteins present in both lanes since they were soluble in different solvents, and additionally, two main phylogenetic groups had resulted from the sequence analysis, therefore it was hypothesized that both findings could be related.

The experimentally detected peptides in MSMS were the queries against the in-house database of protein sequences, which was modified to have 3 different sequences for each protein: the signal peptide, the basic and the acidic subunits. By differentiating the subunits into separate sequences, we avoided the risk of peptides being mistakenly identified between the two subunits, which would result in false positives. This allowed us to more precisely map the peptides considering that the globulin subunits migrated to different positions in the gels, and thus, would be present only in one of the two bands in each lane.

**Figure 4:**
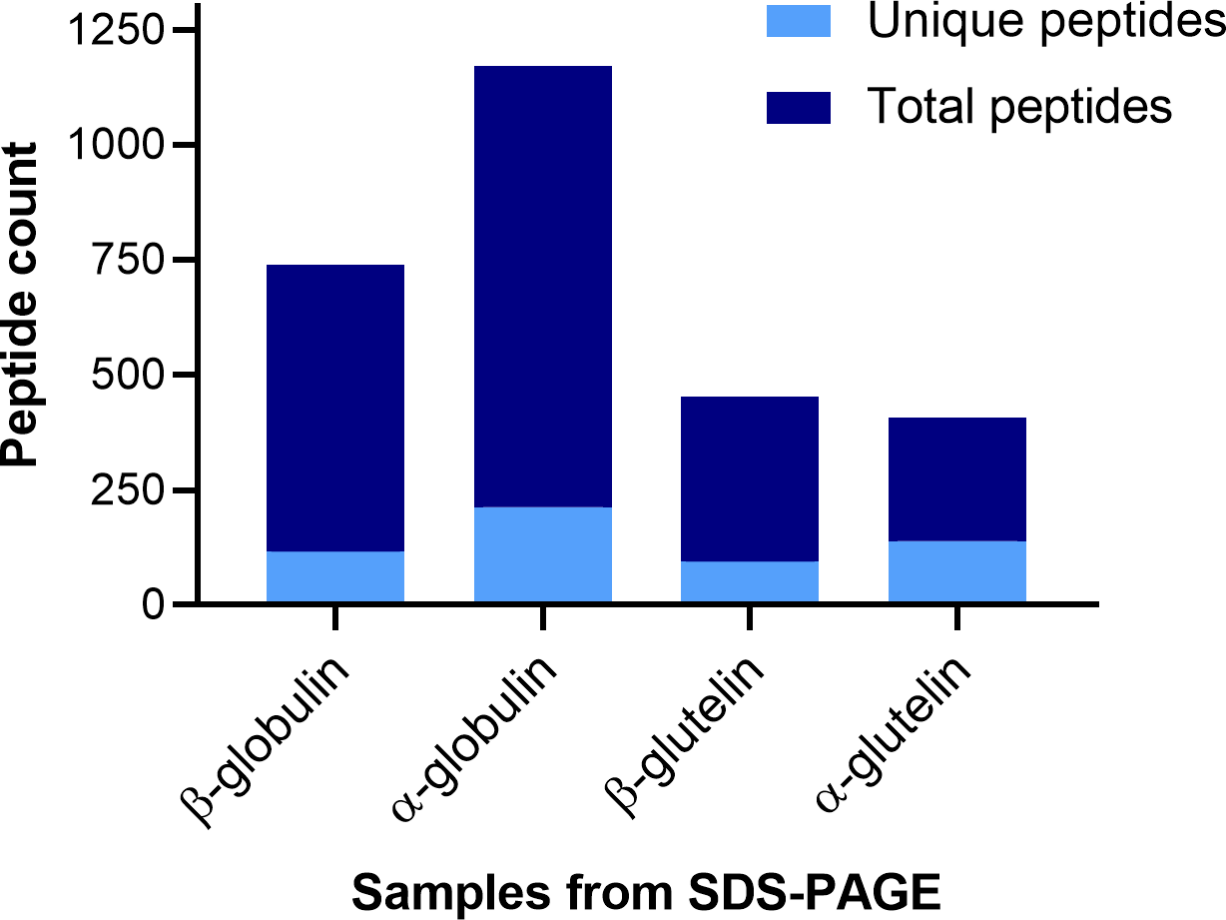
Stacked bar graph of the peptides identified by MSMS from the SDS-PAGE band samples. Representation of the count of total peptides, and the non-duplicated ones (unique), that were identified in each of the protein-containing samples by LC-MSMS (y-axis). The samples refer to protein bands in SDS-PAGE gels: firstly, the globulin subunit that is found at each MW (β or α) and then the gel lane from which the sample was cut (globulin or glutelin).

**Figure 4** represents the total and unique peptide count in each sample. Unique peptides are those that only matched one sequence in the database, therefore confirming the presence of the subunit in the protein pool obtained from the gel band. In the β-subunit globulin sample, there were 738 peptide matches, out of which 116 were non-duplicated. In the α-subunit globulin sample, 1170 matches were found and 213 of these were unique. In the β-subunit glutelin sample, 452 total peptides and 95 unique. In the α-subunit glutelin sample, 408 matches and 139 non-duplicated. The most noticeable observation is that the number of peptides in the globulin lane samples is greater than in the glutelin ones, this was expected since the size and intensity of the bands were visibly different in the gel.

To narrow down the number of peptides obtained and map which proteins are expressed, the unique peptides for each globulin sequence in each of the bands were studied in more detail. The summary is presented in **Table 1**. If a unique peptide was matched to a globulin sequence (rows) it is marked with a cross (X) in the corresponding sample (columns). Out of the 32 sequences from the database, 27 were present in at least one of the 4 samples. Among the 5 globulin sequences with no unique peptides, 3 of them were the low-confidence sequences.

**Table 1:** Presence of unique peptides from each globulin in each sample in MSMS analysis.

	Samples			
	β-globulin	α-globulin	β-glutelin	α-glutelin
**Glo_1**		✕		✕
**Glo_2**				
**Glo_3**	✕	✕	✕	✕
**Glo_4**		✕		✕
**Glo_5**	✕	✕	✕	
**Glo_6**	✕	✕	✕	✕
**Glo_7**	✕	✕	✕	✕
**Glo_8**				
**Glo_9**		✕		
**Glo_10**	✕		✕	
**Glo_11**	✕		✕	
**Glo_12**	✕		✕	
**Glo_13**				
**Glo_14**	✕	✕	✕	✕
**Glo_15**	✕	✕	✕	✕
**Glo_16**	✕	✕	✕	✕
**Glo_17**	✕	✕	✕	✕
**Glo_18**	✕	✕	✕	✕
**Glo_19**		✕		✕
**Glo_20**		✕		
**Glo_21**		✕		
**Glo_22**		✕		✕
**Glo_23**	✕	✕	✕	✕
**Glo_24**		✕		✕
**Glo_25**	✕	✕	✕	✕
**Glo_26**	✕	✕	✕	✕
**Glo_27**	✕	✕	✕	✕
**Glo_28**	✕	✕	✕	✕
**Glo_29**	✕		✕	✕
**Glo_30**		✕		✕
**Glo_31**				
**Glo_32**				

Marked with a cross (✕) is the presence of at least one unique peptide from the MSMS results that matched the in-house globulin-predicted amino acid sequences (shortened with the number, e.g., Glo_1) for each sample (top headers). The samples refer to protein bands in SDS-PAGE gels: firstly, the globulin subunit that is found at each MW (β or α) and then the gel lane from which the sample was cut (globulin or glutelin).

Comparing the bands that migrated to the same MW we observed the same pattern with some exceptions. In the case of the β-subunit bands, the same globulins were found in both, altogether 18. In the case of the α-subunit bands, there are 5 mismatches out of the total of 24 identified globulins between both lanes (**Table 1**).

When comparing the bands from the same lane, more mismatches were detected. As mentioned, in the β-subunit globulin sample, 18 peptides were identified, while the number rises to 23 in the α-subunit globulin sample. Comparing them, for 13 globulin proteins only one of the subunits was detected. In the β-subunit glutelin sample, the same 18 peptides were identified, while in the α-subunit glutelin sample, 20 were detected. Among these 20, in 10 of the proteins, only one of the subunits was found to have unique peptides. The apparent discrepancy between the results within the same gel lane will be discussed afterward.

## 4 Discussion

Oat proteins are among the most nutritious cereal proteins, due to their favorable amino acid composition, which contribute to the intake of essential amino acids from non-animal sources (Leser, 2013; Tang et al., 2023). Based on their biochemical properties, four different oat protein classes can be distinguished regarding their solubility: salt-soluble globulins, water-soluble albumins, alcohol-soluble prolamins, and alkali-soluble glutelins (Spaen and Silva, 2021). In addition to being the most nutritious, the globulins are also the most abundant. Thus, a detailed characterization of all available globulins would be very useful. However, no such comprehensive overview has yet been presented, mainly due to the fact that no complete oat genome sequence was previously available. In this study, thanks to the recent publication of the complete oat genome from the cultivar Sang, we here report the most complete list of globulin genes published in oat, exemplified by the cultivar Belinda. We accomplished this by making a thorough genomic search of globulin-like sequences present on the genome and confirmed the data by expression analysis both at RNA and protein levels using high-resolution MSMS analysis.

By employing EST from 12S globulin genes from published literature we identified 32 sequences in the Sang genome, 29 potential full and 3 partial sequences. Sequence annotations were retrieved from the oat genome. The EST retrieved in the past served as a solid starting point, but the availability of the complete oat genome made it possible to increase the number of full sequences from 16 (Anderson, 2014) to the 29 published in this study. The complexity of the hexaploid oat genome, characterized by multiple repeats and major rearrangements, means we cannot rule out the existence of additional genes that may not yet have been annotated or have lower similarity to those identified (Kamal et al., 2022). These results will now serve as a basis to continue exploring the oat genome and search for even more globulin-like sequences with a lower sequence homology than the ones already retrieved.

When physically localizing the recovered genes in the oat chromosomes we found that only 10% of them were present in the C-subgenome, while the remaining ones were about equally divided among the A and D-subgenomes. These data are in line with the current theory stating that the oat D-subgenome originates from an ancestor A-genome, and therefore nitrogen storage proteins would be found in them since ancient diploid oat species were AA, and they must have had the genetic information to store nitrogen (Yan et al., 2016). It has also been hypothesized that the translocations between the genomes that has occurred through the domestication process also account for the gene loss seen in the C subgenome (Kamal et al., 2022). Thus, this could also add to the fact that fewer globulins are found there.

Multiple sequence alignment of the deduced globulin amino acid sequences revealed a high level of similarity. More than 75% of the residues had a similarity score for their physicochemical properties above 70%. In the phylogenetic analysis two clusters could be distinguished, which is consistent with the previous analysis performed in the CDC Dancer cultivar (Anderson, 2014). Genes from both clusters are present in all three subgenomes. It has been hypothesized that each globulin cluster encodes structurally different globulins, one class originally present in the cell wall and therefore readily solubilized in water and the other in vacuolar protein bodies, which requires salt-solubility (Klose and Arendt, 2012).

The oat flour samples were sequentially treated with different solvents to separate the protein samples, which were visualized in a denaturing polyacrylamide gel. The protein bands observed in the gel correspond to previously observed separation patterns of oat protein extractions (Runyon et al., 2015; Sunilkumar et al., 2017; Sánchez-Velázquez et al., 2021). Regarding globulins, we observed that some were differently extracted in water compared to a low-ionic solvent, again indicating the presence of different classes of globulins with differential solubility (Klose and Arendt, 2012).

The methodology proposed in this study can be used to separate the proteins in oats into broad groups that differ in their solubility. However, it can be improved to obtain cleaner and better-isolated bands in polyacrylamide gel electrophoreses. This will improve the quality of the MSMS samples or any other further analysis that relies on gel separation. Further knowledge about these proteins will allow tailored and more precise isolation based on their physicochemical properties, such as isoelectric points needed to perform two-dimensional gel electrophoresis. This will be especially useful for the water-soluble extracted proteins where a smear still was observed in the experiments performed.

The α- and β-globulin bands were extracted from the globulin and glutelin lanes and were treated with trypsin to obtain the peptide spectrum required for the LC-MSMS separation. In this way we could confirm the physical existence of 27 of the different globulin-predicted sequences identified in the genomic analysis. The four gel samples obtained from the globulin and glutelin lanes allowed us to compare the globulins present in the two different lanes, but also to assess within the same lane whether the subunits detected were the same. These detected peptides originated from sequences within both phylogenetic tree clusters, therefore the reason for having two initial ancestors was not a difference in solubility as initially thought.

As described in the previous section, the comparison between the subunits of the different lanes revealed the detection of the same proteins. This indicates that the globulins extracted in the last step do not belong to a different subgroup. Most likely, they were not extracted with the saline solution because of some structural or physical impediment that needed detergents to be disrupted.

On the other hand, discrepancies between subunits within the same lane are more noticeable, meaning that the presence of both α- and β-subunits could not be verified in some cases. This result is not unexpected since all the proteins share a large part of the sequences and therefore, a higher concentration of the shared peptides and a lower one of the unique parts is expected. Finding a unique peptide is an unequivocal identification of a target protein, however, not finding it for another protein does not mean that it is not present in the sample, it has merely not been detected. The reasons for not detecting a peptide can be numerous: too low concentration, too large or too small peptides, a modification, miscutting, or ion suppression (Li et al., 2010).

Further research to increase the knowledge of oat protein could aim to achieve accurate quantification and characterization. Additionally, efforts should be directed toward identifying novel oat globulins in the genome and studying their functional properties. By addressing these aspects, we can unlock the full potential of oat proteins for enhancing food quality and promoting human health.

## 5 Conclusion

This study presents the most comprehensive set of oat globulin genes, including their amino acid sequences, and expands the current knowledge on these proteins. Overall, our findings suggest that at least 27 different salt-soluble globulin proteins are present and expressed in the oat cultivars Sang and Belinda. The proteins were identified through genomic homology searches followed by protein isolation and MSMS analysis. Future research could explore the existence of additional globulin 12S proteins that are not salt-soluble and that might not be as closely related to the discovered sequences in this article, thereby broadening our understanding of the diversity within this protein family.

## Data availability statement

The original contributions presented in the study are included in the article/**Supplementary Material**. Further inquiries can be directed to the corresponding author.

## Author contributions

AG-G: Investigation, Visualization, Writing – original draft. LS: Conceptualization, Writing – review & editing. KB: Investigation, Resources, Writing – review & editing. OO: Writing – review & editing. JZ: Conceptualization, Resources, Supervision, Writing – review & editing.
