# Supplementary material for: Genomic identification of expressed globulin storage proteins in oat
Source: Front Plant Sci. 2024 Jul 23;15:1418658. doi: 10.3389/fpls.2024.1418658 (PMC11300278; doi:10.3389/fpls.2024.1418658)
Supplement: Supplementary file 1 [file DataSheet_1.docx]

Supplementary Material

# Supplementary Table

**Supplementary Table S1**. Globulin gene sequences identified from the Sang cultivar. For the 32 globulin sequences identified in the published genome for the oat cultivar Sang, it is reported the name given for this study and information on the gene localization (chromosome and start-stop codons), DNA and cDNA length are presented. The amino acid length and protein MW corresponding to protein sequences was determined (header “Protein”).

| **Name** | **Chromosome location**  Start-stop codon | **Gene** | | **Protein** | |
| --- | --- | --- | --- | --- | --- |
|  |  | **DNA length** | **cDNA length** | **Length** | **MW (kDa)** |
| **Globulin 1** | Chr1A  512,247,707-512,249,585 | 1879 | 1539 | 512 | 57.98 |
| **Globulin 2** | Chr1A  512,228, 485-512,230,362 | 1878 | 1539 | 512 | 58.05 |
| **Globulin 3** | Chr1A  512,190,881-512,192,786 | 1906 | 1560 | 501 | 56.52 |
| **Globulin 4** | Chr1A  387,227,076-387,228,952 | 1877 | 1521 | 506 | 57.2 |
| **Globulin 5** | Chr1A  387,185,318-387,187,195 | 1878 | 1536 | 511 | 57.97 |
| **Globulin 6** | Chr1A  373,411,099-373,412,998 | 1900 | 1560 | 519 | 59.02 |
| **Globulin 7** | Chr1A  369,379,519-369,381,418 | 1900 | 1557 | 518 | 58.56 |
| **Globulin 8** | Chr3A  20,658,974-20,660,845 | 1872 | 1527 | 508 | 57.39 |
| **Globulin 9** | Chr4A  326,875,501-326,877,400 | 1900 | 1557 | 518 | 58.67 |
| **Globulin 10** | Chr7A  20,253,880-20,255,744 | 1865 | 1512 | 503 | 56.96 |
| **Globulin 11** | Chr7A  20,313,589-20,315,450 | 1862 | 1509 | 502 | 56.82 |
| **Globulin 12** | Chr7A  40,799,549-40,801,437 | 1889 | 1548 | 515 | 58.06 |
| **Globulin 13** | Chr7A  423,848,846-423,850,647 | 1829 | 1473 | 490 | 55.76 |
| **Globulin 14** | Chr7A  6,922,890-6,924,778 | 1889 | 1548 | 515 | 58.07 |
| **Globulin 15** | Chr2C  426,565,698-426,567,554 | 1857 | 1503 | 500 | 56.78 |
| **Globulin 16** | Chr1C  4,736,571-4,738,454 | 1884 | 1539 | 512 | 57.92 |
| **Globulin 17** | Chr1D  397,120,904-397,122,793 | 1890 | 1548 | 515 | 58.09 |
| **Globulin 18** | Chr1D  377,956,689-377,958,553 | 1865 | 1512 | 503 | 56.83 |
| **Globulin 19** | Chr1D  377,872,367-377,874,228 | 1862 | 1509 | 502 | 56.78 |
| **Globulin 20** | Chr1D  377,751,229-377,753,093 | 1865 | 1512 | 503 | 56.71 |
| **Globulin 21** | Chr1D  351,702,363-351,704,262 | 1900 | 1560 | 519 | 58.82 |
| **Globulin 22** | Chr1D  348,352,182-348,354,081 | 1900 | 1557 | 518 | 58.63 |
| **Globulin 23** | Chr3D  432,320,74-432,322,602 | 1863 | 1509 | 503 | 56.9 |
| **Globulin 24** | Chr3D  453,231,739-453,233,713 | 1975 | 1584 | 527 | 59.3 |
| **Globulin 25** | Chr4D  310,345,162-310,347,061 | 1900 | 1557 | 518 | 58.54 |
| **Globulin 26** | Chr4D  310,374,644-310,376,543 | 1900 | 1557 | 518 | 58.61 |
| **Globulin 27** | Chr7D  322,572,848-322,574,712 | 1865 | 1509 | 502 | 57.08 |
| **Globulin 28** | Chr1A  1,230,571-1,232,443 | 1872 | 1584 | 527 | 59.36 |
| **Globulin 29** | Chr3D  447,835,515-447,837,415 | 1901 | 1548 | 515 | 58.01 |
| **Globulin 30** | Chr3D  447,845,627-447,847,497 | 1871 | 1518 | 505 | 56.92 |
| **Globulin 31** | Chr7C  213,497,176-213,498,967 | 1792 | 1419 | 472 | 53.35 |
| **Globulin 32** | Chr1D  370,393,694-370,395,331 | 1955 | 1431 | 476 | 53.44 |

# Supplementary Figures


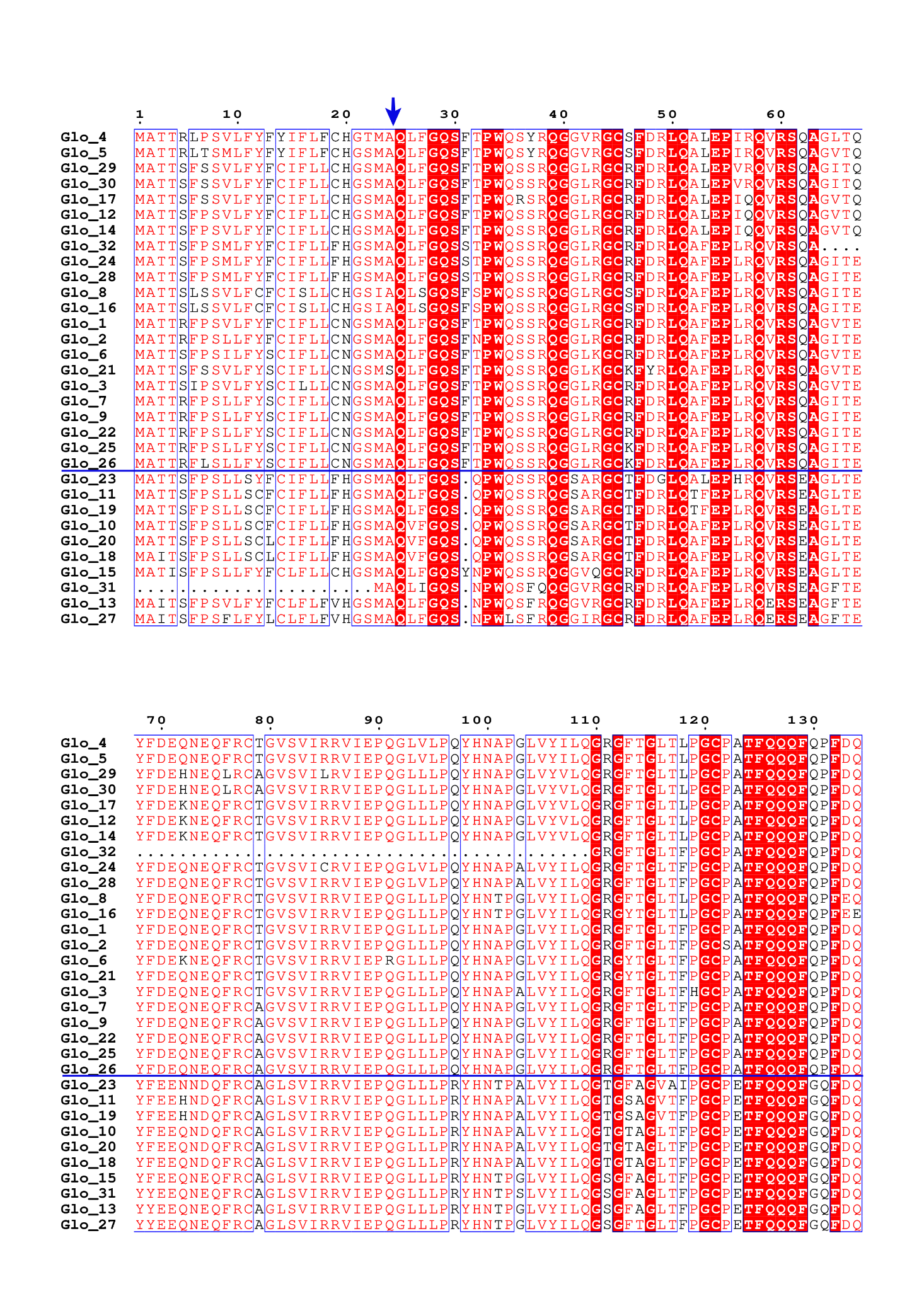


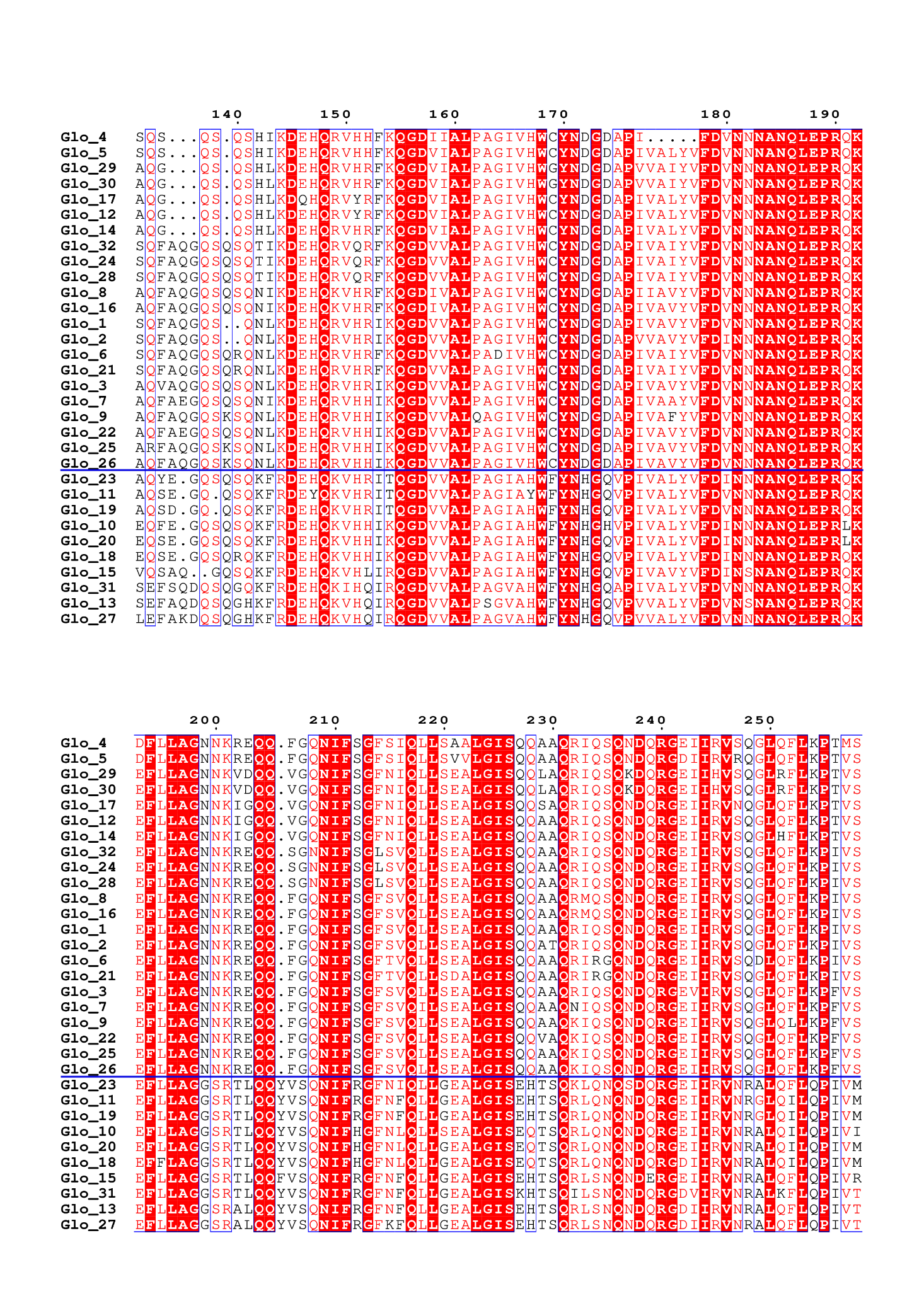

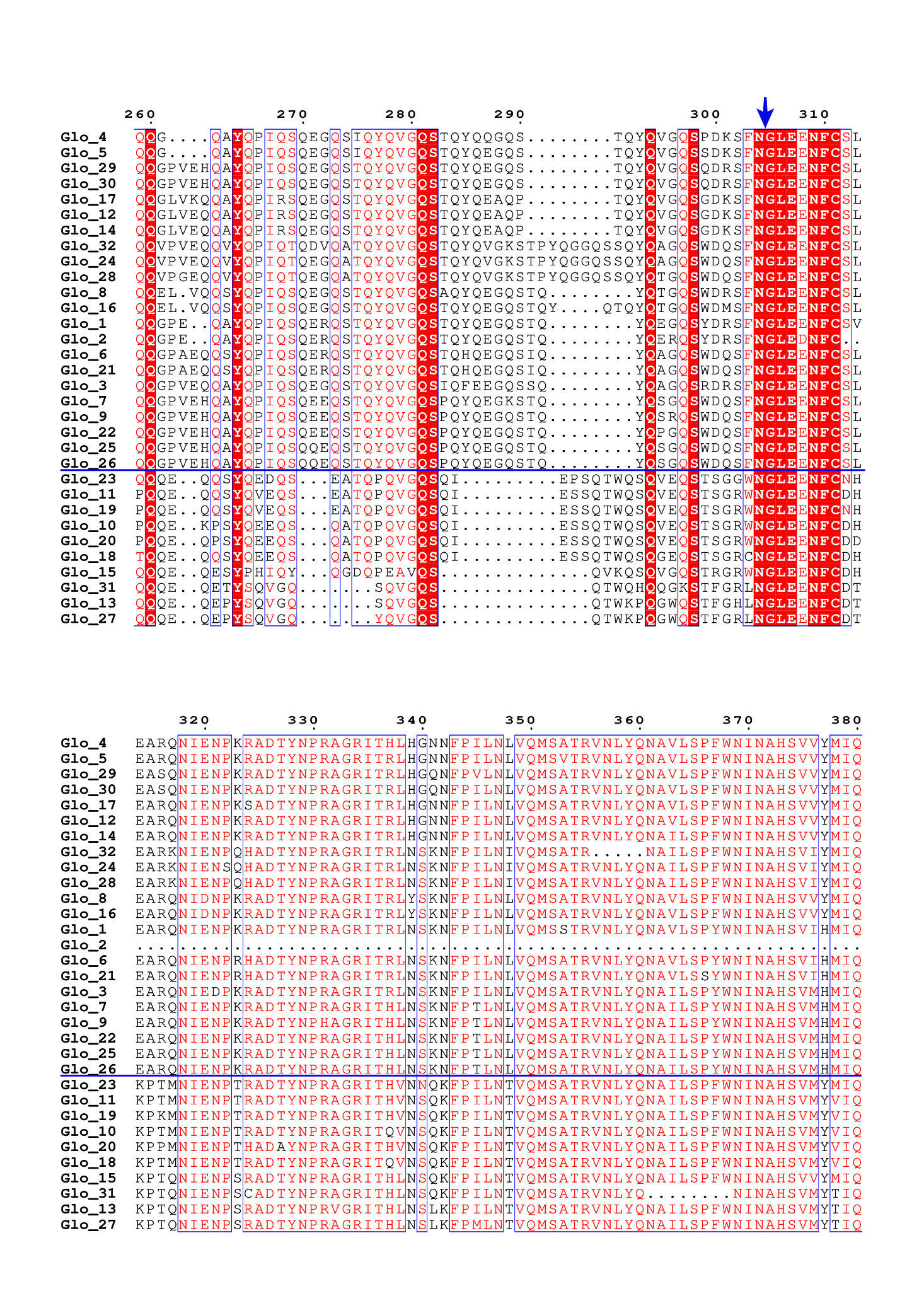

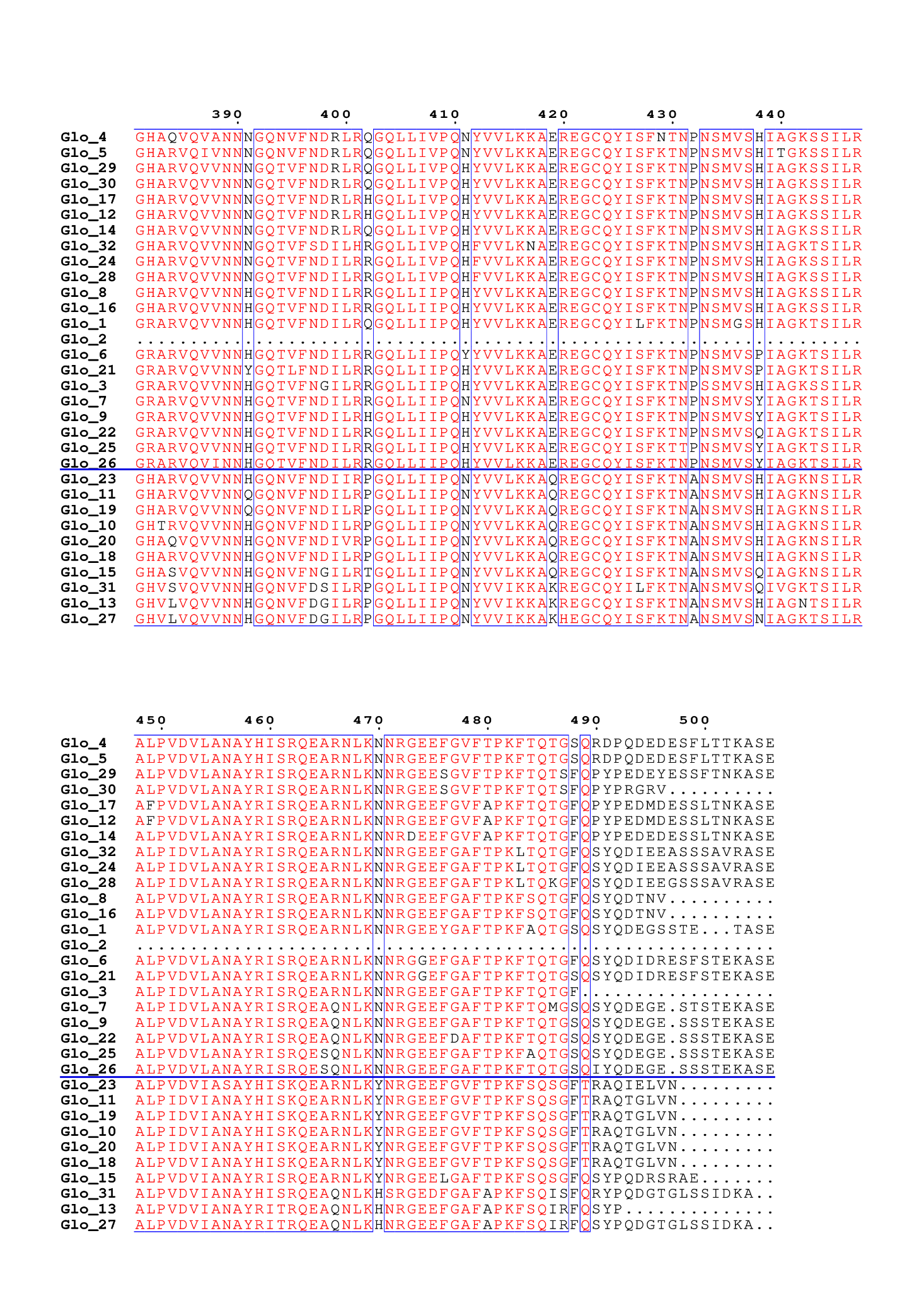


**Supplementary Figure S1. Multiple sequence alignment of the 32 globulin protein sequences**. The MSA was performed with Clustal Omega and the visualization with ESPripr 3.0 (Sievers *et al.*, 2011; Robert and Gouet, 2014). Residues in red and framed in blue have a similarity score for the column based on physicochemical properties higher than 70 %. Strict identity is represented with white residues on a red background. Blue arrows indicate site of cleavage for the signal peptide (position 24) and for the two polypeptides (position 304). The blue horizontal line between globulin 26 and 23 separates the two main phylogenetic clusters.


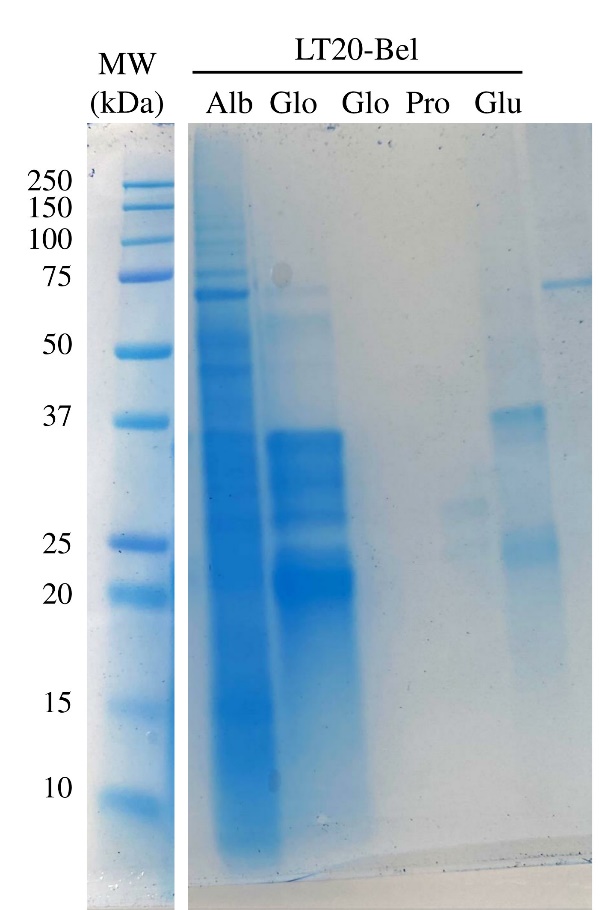

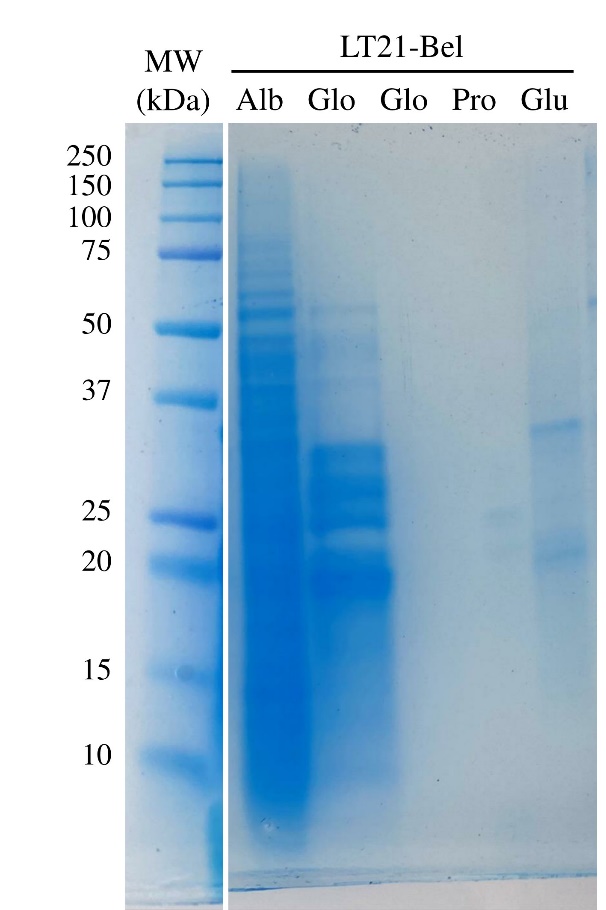


**Supplementary Figure S2. SDS-PAGE analysis of protein extracted from oat flour with a duplicated step**. Oat proteins fractions (albumin – Alb, globulin – Glo, prolamin – Pro, glutelin – Glu) were extracted by employing solvent solubilization. Samples from the Belinda cultivar and two different harvests were used (LT-20 Bel, left panel, and LT21-Bel, right panel). The protocol for the extraction of globulin was duplicated. The gel used had a 8–16% polyacrylamide gradient and was stained with Coomassie. Molecular weight marker is shown as kDa on the left side.


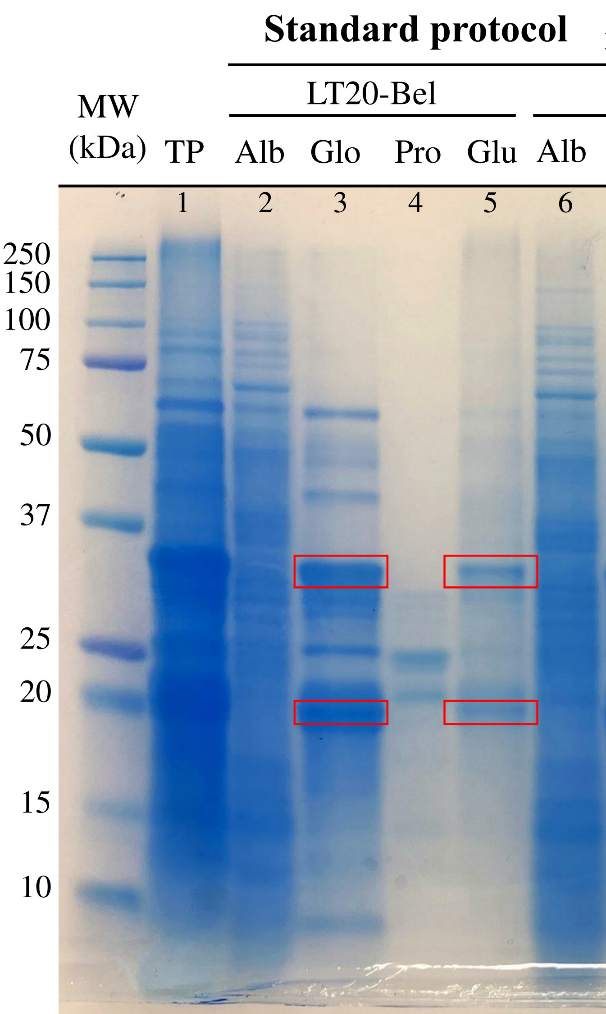


**Supplementary Figure S3. Selected gel bands for MSMS analysis.** Framed in red are the bands that were cut from the gel. They were named after the globulin subunit that is found at each MW (β or α) and then the gel lane from which the sample was cut (globulin or glutelin).
